# Psychometric properties of the persian version of the physician teaching self-efficacy questionnaire

**DOI:** 10.1186/s12909-023-04130-6

**Published:** 2023-03-15

**Authors:** Ali Asghar Hayat, Karim Shateri, Sepideh Kamalian Fard, Elnaz Sabzi Shahr Babak, Hatam Faraji Dehsorkhi

**Affiliations:** 1 Clinical Education Research Center, Faculty of Medicine, Shiraz University of Medical Sciences, Shiraz, Iran; 2 Department of Primary Education, Abdanan Center, Islamic Azad University, Abdanan, Iran; 3 Shiraz University of Medical Sciences, Shiraz, Iran; 4 Educational Management, Shahid Bahonar University, Kerman, Iran

**Keywords:** Teaching self-efficacy, Motivation, Clinical teaching, Psychometric properties

## Abstract

**Introduction:**

Theories and numerous empirical studies indicate teaching performance and students’ learning progress are affected by teaching self-efficacy. Therefore, the present study examines the psychometric properties of the Persian version of the physician teaching self-efficacy questionnaire.

**Methods:**

The 16-item physician teaching self-efficacy questionnaire was translated from English to Persian and back-translated to English and then administered to 242 medical teachers from six medical universities. To assess construct validity, researchers made use of confirmatory factor analysis. To check the reliability and validity of the physician teaching self-efficacy questionnaire, we used internal consistency, discriminant, convergent, and criterion validity.

**Results:**

PLS-SEM results substantiated the original three factor structure of the questionnaire which is dyadic, triadic, and self-regulation. For all sub-scales, internal consistency- measured by Cronbach’s alpha and composite reliability, convergent validity- measured by factor loading and AVE, and discriminant validity- measured by cross-loading, Fornell-Larcker, and HTMT metrics- confirmed the construct reliability and validity of the questionnaire. A positive correlation was, also, fund between teaching motivation and experience with the physician teaching self-efficacy questionnaire scales, proving the criterion validity of the questionnaire.

**Conclusion:**

The Persian version of physician teaching self-efficacy questionnaire is a valid, highly reliable, and multidimensional tool to measure physicians’ clinical teaching self-efficacy working in medical universities.

## Introduction

Teaching, as one of the essential roles and responsibilities of medical teachers [1], is a complex, challenging, demanding, and highly unpredictable task [2, 3]. Higher education developments such as demands for accountability, quality assurance and shifts from teacher-centered approaches to learner-centered ones [4, 5] have made the teaching profession even more demanding, challenging, and uncertain. This circumstance requires physicians to not only possess knowledge and skills but also believe in their capacities to cope with demands, address challenges and difficulties, and handle uncertainties along the teaching process- which is called teaching self-efficacy.

Teaching self-efficacy, conceptualized as medical teachers’ confidence, trust, or beliefs in their instructional capabilities to deliver high-quality teaching [6, 7], is a game changer, making a real difference in teaching and learning. It is proven to influence instruction, teachers, and students [6].

With regard to instruction, self-efficacy affects pre-teaching activities (such as lesson plan development), in-teaching activities (such as effective classroom management, supportive classroom climate, and cognitive activation) [6], and post-teaching activities (such reflection on teaching) [7]. In fact, it is demonstrated to be a long-term determinant of teaching quality [6, 8, 9]. With regard to teachers, self-efficacious teachers demonstrate high job satisfaction [10, 11], better well-being [12–14], more commitment [15, 16], and effectiveness [17]. Concerning students, teaching self-efficacy is proven to contribute to students’ academic achievement [18–20], and motivation [21]. In this regard, in a meta-analysis study, it was demonstrated that self-efficacy of teachers is associated to academic achievement of students [22].

In settings other than higher education, self-efficacy has been empirically proven to be related to performance [23]. In fact, several meta-analyses have substantiated the effect of self-efficacy on job performance [24–26]. In higher education, in general, and medical education, in specific, however, teaching self-efficacy is under researched [2, 27, 28].

Therefore, research into teaching self-efficacy is of significance to capture a better understanding of the phenomenon. To measure self-efficacy of teaching in medical setting, Dybowski, Kriston, and Harendza (2016) developed and tested the physician teaching self-efficacy questionnaire (PTSQ) [7]. The results validated the instrument, indicating its suitability to assess physicians’ self-efficacy of teaching. PTSQ being specific to medical context is considered its merit, helping determine whether and to what degree training, as a solution, is needed to promote medical teachers’ teaching self-efficacy. As suggested by authors, however, PTSQ is needed to be tested in different languages and cultures to establish its value. Taking the advice, the present study is an endeavor to check the psychometric properties of PTSQ to determine whether it is a suitable tool for assessing teaching self-efficacy of physicians in Iran. Any attempt of this sort is of great value, especially in developing countries like Iran since universities suffer from a shortage of resources while teaching staff are expected to live up to international academic standards.

## Methods

### Participants and setting

The present study involved a cross-sectional research design. The target population included all the medical teachers form 6 universities. To determine the sample size, the 10 times rule was the method of choice. This rule, which is well suited for PLS-SEM, indicates minimum 10 cases per indicator [29, 30]. Hence, a convenient sample of 395 was selected which is well above the sample size recommended by the rule to avoid low response rate. Being a full-time physician, being willing to participate, and being involved in bedside teaching were considered as the inclusion criteria. The sample was, then, asked to complete the PTSQ and the physician teaching motivation questionnaire (PTMQ) on a 5 point Likert scale ranging from 1 = strongly disagree to 5 = strongly agree. Of 395, 257 questionnaires were returned, yielding a response rate of 65%. Nonetheless, 15 of the returned questionnaires were discarded over incompleteness. Ultimately, 242 returned questionnaires were eligible for analysis.

### Procedure

We employed the PTSQ, which is a valid, reliable, and self-expressed 16 item questionnaire, to assess medical teachers’ teaching self-efficacy [7]. The PTSQ reflects medical educators’ beliefs that they can provide high-quality clinical instruction even when faced with frequently occurring critical teaching situations such as patient selection, related problems, time constraints, allocating insufficient time to teach, disruptions of the lessons, or unmotivated learners [31].

Based on the guidelines established by Brislin (1970) [32] and Jones et al.(2001) [33], for the translation and adaption of research instruments, the PTSQ was translated into Persian. So the first two bilingual experts in English and Persian from medicine and medical education disciplines translated and edited this questionnaire. A panel of experts, including four faculty members (from the social medicine and cardiology, educational psychology, and medical education departments), reviewed the Persian translation, and based on their recommendations, it was updated to maintain translation quality. This was then translated back by two independent, bilingual experts ((lacking access to the original form). Then, two bilingual and independent experts provided the back translation (lacking access to the original form). After comparing the two versions with one another and discussing any differences by two experts who were proficient in English, they agreed on the final version. Finally, results from pilot testing revealed that eight medical teachers had no trouble comprehending and completing the questionnaire.

Quantitative and qualitative means were deployed to check the content validity of the measure. As mentioned earlier, a panel of 6 experts were first asked to assess each item in terms of grammar, comprehensibility, wording, item allocation, as well as scaling. Some items were modified based on the experts’ inputs. Afterwards, 8 medical teachers were requested to quantitatively examine measure’s content validity using I-CVI and S-CVI methods. The technique offered by Waltz and Bausell was used for content validity index (CVI) assessment [34, 35]. Thus, the panel rated each item in terms of relevance, clarity, and simplicity on a 4-point scale. In CVI assessment, the critical value of 0.79 is deemed acceptable [35]. At first, the research put together a validation form to ensure that the panel had the right expectations and understanding of the task. Therefore, the researchers provided the panel with information on the definitions, research objectives, conceptual framework, and domain of the measure to medical teachers.

Also, to check criterion validity, we calculated the correlation coefficient of all teaching self-efficacy subscales with teaching motivation and teaching experience.

The adjusted Persian version of PTSQ was administered in six different universities, including; Kerman, Shiraz, Isfahan, Tehran, Jahrom, and Kashan. In each university, an assistant researcher was used to collect data while providing permission from the National Ethics Committee. We performed a confirmatory factor analysis to assess the construct validity of the three PTSQ subscales and to compare the Persian version’s similarity to the English version’s original hypothesized measurement model.

### Tools and materials

#### Physician teaching self-efficacy questionnaire (PTSQ)

The original 16 item PTSQ was developed and validated by Dybowski, Kriston, and Harendza [7], and demonstrated to be comprised of the following three sub-scales which improves teaching-learning process:

Self-regulation involves dealing with challenges facing a teacher during teaching. Sample self-regulation item is “*Even if I am in a bad mood or feel stressed, I give a good lesson.*”

Dyadic regulation, which entails addressing challenges involved in teacher-student relationship. Sample dyadic regulation item is “*I am able to integrate even the weakest students into the lesson.*”

Triadic regulation involves dealing with challenges stemming from interactions between teacher, student, and patient. Sample triadic regulation is “*Even if a patient shows a difficult conduct, I provide a good lesson*.”

To rate the responses a five-point Likert scale ranging from 1 to 5 was used.

#### Physician teaching motivation questionnaire (PTMQ)

According to Banduar’s social cognitive theory [36], self-efficacy is a motivational construct affecting one’s readiness, persistence, and accomplishment. Research shows that self-efficacy and motivation are inter-related and powerfully predict one another [37, 38]. SO, we supposed teaching motivation and self-efficacy are positively associated.

The PTMQ is a valid and reliable questionnaire to assess the Physician’s teaching motivation, developed by Dybowski and Harendza [39]. It includes a 5-point Likert scale with the following five subscales: intrinsic motivation (sample item: *I enjoy my teaching most of the time*), identified motivation (sample item: *I teach because I find my lessons’ contents important*), introjected motivation (sample item: *I teach because otherwise I would have a bad conscience towards my colleagues*), external motivation (sample item: *I mainly teach because it belongs to my scope of duties)*, amotivation *(*sample item: *I teach although I hardly ever feel like doing it).*

The validity and reliability of the PTMQ were approved by Dybowski and Harendza [39]. Besides, good internal consistency was obtained in our study as well (Cronbach’s α = 0.88).

### Teaching experience

Based on social cognitive theory, enactive mastery experiences is one of the most significant sources of self-efficacy, instances in which a person feels successful in completing a task [40]. Therefore, as in previous research [7], we supposed teaching experience and self-efficacy are positively associated. As for demographic information, we collected data on age, sex, city of work, occupational position, academic rank, and years of teaching experience.

### Data analysis

To determine the validity and reliability and assess the measurement model of PTSQ, we applied Smart-PLS 3. The data were analyzed according to the steps suggested for the evaluation of reflective measurement models where item loadings, internal consistency reliability, discriminant and convergent validity are checked respectively [41–44]. Loadings higher than 0.70 are recommended because they adequately explain about 50% of the variance of the indicators and, hence provide satisfactory item reliability [44].

To evaluate internal consistency reliability, researchers made use of composite reliability (CR) and Cronbach’s alpha (α). CR represents a more accurate measure of reliability since the items are weighted relying on the indicators’ independent loadings [44]. CR and α values are acceptable if higher than 0.7 [45]. According to Hair et al., CR values between 0.70 and 0.90 are regarded as satisfactory to good [44].

To evaluate the latent variables convergent validity, subsequently, researchers made use of average variance extracted (AVE) which should be equal or higher than 0.5. [44, 45]. Discriminant validity reveals the degree of difference of a given latent variable from other latent variables [44, 46]. Cross-loadings, Fornell-Larker criterion, and the Heterotrait-Monotrait ratio (HTMT) are recommended to assess discriminant validity [47]. Cross-loadings are grounded on the assumption that the items should exhibit the highest association with their respective latent variable in comparison to other latent variables. According to Fornell-Larcker criterion, the latent variables square root of AVE must be larger than the correlation of that variable with other latent variables [45]. To test discriminant validity, the Heterotrait-Monotrait ratio (HTMT) has been recently suggested as an important measure, which assesses the average of the Heterotrait– hetero method correlations when high values of HTMT are observed, discriminant validity problems appear. In this regard, Hensler et al. (2015) suggested a criterion of 0.90 [48]. Finally, to check criterion validity, we calculated the correlation coefficient of all teaching self-efficacy subscales with teaching motivation and teaching experience using Pearson’s correlation coefficient.

### Ethical considerations

In the current study, we first obtained the approval of the university’s ethics committee (IR.SUMS.REC.1398.435) and then informed consent forms were completed by participants. Also, we distributed and collected anonymous questionnaires among the participants.

## Results

As previously told, 242 questionnaires were included for analysis. The participants’ demographic characteristics are indicated in Table 1.

**Table 1:** The participants’ demographic profile

Variable	Group	N	%
Sex	Female	105	43.4
	Male	125	51.7
	Missing	12	5.0
Age	30 years and less	13	5.4
	31 to 40 years	72	29.8
	41 to 50 years	77	31.8
	51 years and older	53	21.9
	Missing	27	11.2
City	Jahrom	42	17.4
	Shiraz	39	16.1
	Isfahan	55	22.7
	Tehran	36	14.9
	Kerman	32	13.2
	Kashan	38	15.7
Rank	Instructor	23	9.5
	Assistant Professor	122	50.4
	Associate professor	37	15.3
	Full professor	25	10.3
	Missing	35	14.5
Years of experience	1 to 10	117	48.3
	11 to 20	63	26.0
	21 and older	33	13.6
	Missing	29	12

### Content validity results

Based on the ratings of each of the 16 items by the 8 experts, Table 2 shows I-CVI of the items of the constructs and the aggregate mean I-CVI. The aggregate mean CVI as computed is greater than 0.79 for relevance (= 0.92), clarity (= 0.96), and simplicity (= 0.98), demonstrating that the questionnaire is content-valid.

**Table 2:** I-CVI and S-CVI of scale by 8 experts

Construct	Item	I-CVI For Relevance	I-CVI For Clarity	I-CVI For Simplicity
Self- regulation	q1	0.87	1	1
	q2	0.87	1	1
	q3	0.87	0.87	0. 87
	q6	1	1	1
	q7	1	0.87	1
	q11	0.87	1	1
Dyadic regulation	q4	0.87	1	1
	q5	1	1	1
	q8	0.87	1	1
	q9	1	0.87	1
	q10	0.87	1	1
Triadic regulation	q12	1	1	0.87
	q13	0.87	1	1
	q14	0.87	1	1
	q15	1	0.87	1
	q16	1	1	1
Scale-level Content Validity Index	S-CVI = 0.92		S-CVI = 0.96	S-CVI = 0.98

We calculated inter-correlations of all scales. The triadic regulation and self-regulation subscales exhibited the highest and lowest association with the aggregate score, respectively. In addition, all subscales showed high associations with each other (Table 3).

**Table 3:** The correlation matrix findings

Scales	TSE	SR	TR	DR
Teaching self-efficacy	1			
Self-regulation	0.87	1		
Triadic regulation	0.89	0.68	1	
Dyadic regulation	0.88	0.62	0.71	1

Note: DR: Dyadic regulation; TR: Triadic regulation; SR: Self-regulation; TSE: Teaching self-efficacy

The results are reported based on the steps suggested for the evaluation of reflective measurement models by Hair et al. [44] where item loadings, internal consistency reliability, discriminant and convergent validity are investigated respectively.

### Examining the indicator loadings

**Fig. 1:**
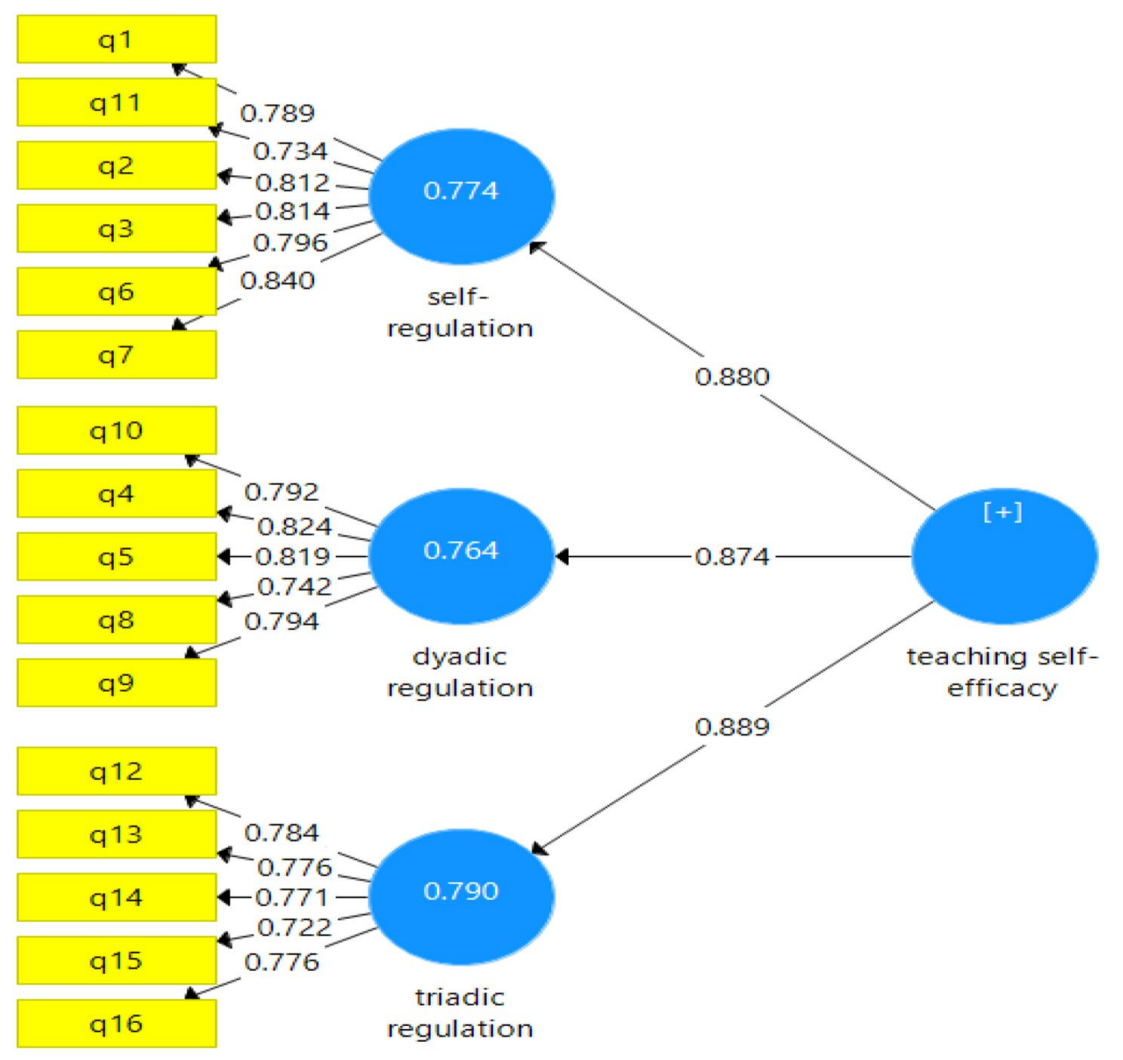
Confirmatory factorial analyses for the Persian version of physician teaching self-efficacy

Loadings higher than 0.70 are recommended because they adequately explain about 50% of the variance of the indicators and hence provide satisfactory item reliability [44]. The confirmatory factor analysis results showed the item loadings were between 72 and 84 (Fig. 1; Table 4); therefore, it can be concluded that the observable variables in this study demonstrate appropriate reliability.

### Assessing internal consistency reliability

Based on findings, Cronbach’s alpha for self-efficacy, self-regulation, triadic regulation, and dyadic regulation, was revealed to be 0.92, 0.88, 0.82, and 0.85, respectively, demonstrating a strong internal consistency among items. In addition, the results revealed teaching self-efficacy scale and its subscales’ CR scores were higher than 0.7, indicating the scales’ high reliability applied in the present research (Table 4).

### Examining the convergent validity

As indicated in Table 4, teaching self-efficacy and its subscales retained appropriate AVE ranging from 0.59 to 0.64, which passed the suggested criterion of 0.5.

**Table 4:** Factor Loadings, CR, and AVE (n = 242)

Construct	Questions	Loadings	(α)	(CR)	AVE
Self- regulation	q1	0.79	0.88	0.91	0.64
	q2	0.81			
	q3	0.81			
	q6	0.80			
	q7	0.84			
	q11	0.73			
Dyadic regulation	q4	0.82	0.85	0.89	0.63
	q5	0.81			
	q8	0.74			
	q9	0.79			
	q10	0.79			
Triadic regulation	q12	0.78	0.82	0.87	0.59
	q13	0.77			
	q14	0.77			
	q15	0.72			
	q16	0.78			
Teaching self-efficacy total			0.92	0.93	0.62

### Discriminant validity

As mentioned, the Cross-loadings, Fornell-Larker criterion, and the Heterotrait-Monotrait ratio (HTMT) are recommended to assess discriminant validity. According to Table 5, it is comprehensible that all 16 questions demonstrate the highest correlation with their latent variable as opposed to other latent variables, and therefore it can be asserted that the cross-loadings criterion has been achieved.

**Table 5:** Cross-loading analysis

	Self- regulation	Dyadic regulation	Triadic regulation
item_1_	**0.79**	0.44	0.47
item _2_	**0.81**	0.45	0.46
item _3_	**0.81**	0.53	0.52
item _4_	0.49	**0.82**	0.56
item _5_	0.53	**0.82**	0.59
item _6_	**0.80**	0.59	0.61
item _7_	0.84	0.50	0.55
item _8_	0.51	**0.74**	0.57
item _9_	0.48	**0.79**	0.59
item _10_	0.41	**0.79**	0.54
item _11_	**0.73**	0.41	0.54
item _12_	0.48	0.53	**0.78**
item _13_	0.49	0.58	**0.77**
item _14_	0.50	0.55	**0.77**
item _15_	0.51	0.63	**0.72**
item _16_	0.49	0.49	**0.78**

In the following, as revealed in Table 6, the square roots of AVE were larger than the inter-correlation between the research constructs; therefore, findings demonstrated an acceptable discriminant validity.

**Table 6:** Fornell-Larcker Criterion Results

Construct	Self- regulation	Dyadic regulation	Triadic regulation
Self- regulation	**0.80**		
Dyadic regulation	0.61	**0.79**	
Triadic regulation	0.66	0.71	**0.77**

As indicated in Table 7, the HTMT ratios are less than 0.90, which means there is no problem with construct’s discriminant validity. Thus, the HTMT ratio criterion is fulfilled in the present study.

**Table 7:** HTMT Discriminant validity

Construct	1	2	3
1-Self- regulation	-		
2-Dyadic regulation	0.70	-	
3-Triadic regulation	0.77	0.83	-

### Concurrent criterion validity

As indicated in Table 8, self-efficacy of teaching and its subscales revealed positive and significant correlation with identified teaching motivation and intrinsic teaching motivation, respectively. Moreover, self-efficacy of teaching and all its subscales indicated the largest negative association with teaching amotivation and then external and introjected teaching motivation. In addition, all teaching self-efficacy scales showed a positive and significant association with teaching experience. Based on Table 8, among self-efficacy subscales, self-regulation and dyadic showed the highest and the lowest correlations accordingly.

**Table 8:** Correlations of the teaching self-efficacy with teaching experience and motivation

	Teaching self-efficacy total	Self- regulation subscale	Dyadic subscale	Triadic subscale
Intrinsic motivation	0.32**	0.26**	0.29**	0.31**
Identified motivation	0.55**	0.48**	0.49**	0.53**
Introjected motivation	-0.19**	-0.20**	-0.15*	-0.17**
External motivation	-0.19**	-0.22**	-0.18**	-0.20**
Amotivation	-0.27**	-0.24**	− 0.026**	− 0.021**
Teaching experience	0.30**	0.33**	0.21**	0.28**

## Discussion

This study attempted to validate the PTSQ in the Persian context. The confirmatory factor analysis findings supported the PTSQ three-factor structure in Persian context, as reported in original version [7]. The findings showed that the Persian version of PTSQ has acceptable psychometric properties to be used among Iranian physicians, based on obtained indicator loadings, internal consistency reliability, and construct validity. All 16 PTSQ questions assessed their respective latent constructs well with significant loadings which confirms the previous research findings in which the factorial validity of three-factor model has been supported.

The confirmatory factor analysis findings revealed all factor loadings of observable variables were larger than the threshold of 0.70 (0.72 to 0.84), indicating an appropriate item reliability [44]. In previous study conducted in German context, the factor loadings of observable variables were also at an acceptable level [7] which supports the results of the present study.

Acceptable findings were also obtained regarding the reliability of the studied questionnaire. The findings evidenced that the Persian PTSQ total score maintains a remarkable internal consistency (α = 0.92) (Table 2). Also, findings showed that dyadic regulation, self-regulation, and triadic regulation subscale held a good internal consistency. According to the retained results, self-regulation retained the highest (a = 0.88) and triadic regulation (a = 0.82) maintained the lowest internal consistency. In a research conducted by Dybowski et al. (2016), it was found that PTSQ have an excellent internal consistency (α = 0.90). Moreover, their findings showed PTSQ subscales have a good to acceptable consistency (0.85 for self-regulation, 0.77 for dyadic regulation and 0.79 for triadic regulation) [7]. Also, the results of CR were proof of the excellent internal consistency of the questionnaire (0.87 to 0.93). Furthermore, findings indicated among Persian PTSQ subscales, dyadic regulation and self-regulation held the lowest and the highest association with the total score (0.88 and 0.85) accordingly. In Dybowski et al. (2016) study it was discovered that self-regulation and triadic regulation maintained the highest and the lowest correlation with the total score of PTSQ [7].

The results of AVE, used as a criterion to evaluate the construct convergent validity [44], revealed that the Persian PTSQ and its components retained an acceptable convergent validity that exceeded the threshold of 0.50 [44]. Cross-loadings was used to measure item-level discriminant validity [48] and the results proved that each of the questions was differentially loaded on its associated latent variable, indicating the fulfillment of the cross loadings criterion. The results of Fornell-Larker criterion, used to evaluate latent variables discriminant validity [49], demonstrated the latent variables are well distinct from each other. The findings also showed that the HTMT ratio is less than a threshold value of 0.90 [48], indicating there is no problem with constructs discriminant validity.

To evaluate concurrent criterion validity the researchers made use of teaching motivation questionnaire [39]. The results showed that the Persian PTSQ and its subscales were positively and significantly correlated to identified teaching motivation and intrinsic teaching motivation, respectively. Also, the Persian PTSQ and its subscales showed the most negative correlation with teaching amotivation and then external and introjected teaching motivation. In this regard, a similar result has been obtained in previous research [7].

In addition, regarding the relationship between the Persian PTSQ and its subscales with teaching experience, the findings showed that the Persian PTSQ and its subscales were positively and significantly correlated with teaching experience. Previous research has shown that the longer a teacher’s years of teaching, the higher their teaching self-efficiency [19, 50–52]. In a study it was discovered teachers holding more teaching experience years retained higher levels of efficacy [19]. Likewise, Cheung (2008) proved longer teaching experience is an important predictor of higher teacher efficacy [51]. Teacher’s eagerness to teach and their self-efficacy beliefs are affected by teaching experience [53] and it can be said that the more teachers interact with students and their parents, the more their self-efficacy skills grow over time [54]. Of course, the findings on the association between self-efficacy and teaching experience are contradictory. For example, in a study conducted by Guo et al. [55], it was found that teachers’ self-efficacy and their teaching experience years were negatively associated. In this regard, it can be said that increasing teaching years does not necessarily mean increasing teacher teaching skills, and naturally there are teachers who do not grow in terms of teaching quality and teaching skills as their years of service increase.

### Limitations

As in all scientific studies, the present study has some limitation. The first limitation of the current study was that only Physicians completed the Persian version of PTSQ was completed only by physicians. The results aren’t, hence, applicable to other health-related disciplines. Also, in this study, we used a cross-sectional, self-reported data that carries the risk of common method variance (CMV). Moreover, individuals participated in this study voluntarily and naturally; therefore, this study is not free from self-selection bias because it is possible that these people are more motivated and self-efficient.

### Future research

It is recommended that the PTSQ be translated into other languages and tested in other cultures to determine its applicability in various contexts. In this research, our data regarding the PTSQ was acquired through medical teachers self-reporting; therefore, it is suggested to use other data sources like students or other methods that provide more objective data in future researches. In the present research, researchers made use of teaching motivation and teaching experience to check concurrent criterion validity; therefore, it is recommended to use other variables that can be theoretically related to teaching self-efficacy in future researches.

## Conclusion

The results of the current study revealed that the Persian Version of the Physician Teaching Self-Efficacy Questionnaire (PTSQ) retains high reliability and good validity in the Iranian context which supports its possible use in a different national setting. This study can help other researchers interested in researching physician teaching self-efficacy in the Iranian context.

## Data Availability

The datasets generated and/or analyzed during the current study are not publicly available due to privacy and ethical considerations but are available from the corresponding author on reasonable request.
